# MAPK Target Sites of Eyes Absent Are Not Required for Eye Development or Survival in *Drosophila*

**DOI:** 10.1371/journal.pone.0050776

**Published:** 2012-12-12

**Authors:** Barbara Jusiak, Abuduaini Abulimiti, Nele Haelterman, Rui Chen, Graeme Mardon

**Affiliations:** 1 Program in Developmental Biology, Baylor College of Medicine, Houston, Texas, United States of America; 2 Human Genome Sequencing Center, Baylor College of Medicine, Houston, Texas, United States of America; 3 Department of Molecular and Human Genetics, Baylor College of Medicine, Houston, Texas, United States of America; 4 Department of Pathology, Baylor College of Medicine, Houston, Texas, United States of America; 5 Department of Neuroscience, Baylor College of Medicine, Houston, Texas, United States of America; 6 Department of Ophthalmology, Baylor College of Medicine, Houston, Texas, United States of America; 7 Program in Cell and Molecular Biology, Baylor College of Medicine, Houston, Texas, United States of America; Indiana University, United States of America

## Abstract

Eyes absent (Eya) is a highly conserved transcription cofactor and protein phosphatase that plays an essential role in eye development and survival in *Drosophila*. Ectopic eye induction assays using cDNA transgenes have suggested that mitogen activated protein kinase (MAPK) activates Eya by phosphorylating it on two consensus target sites, S402 and S407, and that this activation potentiates the ability of Eya to drive eye formation. However, this mechanism has never been tested in normal eye development. In the current study, we generated a series of genomic rescue transgenes to investigate how loss- and gain-of-function mutations at these two MAPK target sites within Eya affect *Drosophila* survival and normal eye formation: *eya^+^GR*, the wild-type control; *eya^SA^GR*, which lacks phosphorylation at the two target residues; and *eya^SDE^GR*, which contains phosphomimetic amino acids at the same two residues. Contrary to the previous studies in ectopic eye development, all *eya* genomic transgenes tested rescue both eye formation and survival equally effectively. We conclude that, in contrast to ectopic eye formation, MAPK-mediated phosphorylation of Eya on S402 and S407 does not play a role in normal development. This is the first study in *Drosophila* to evaluate the difference in outcomes between genomic rescue and ectopic cDNA-based overexpression of the same gene. These findings indicate similar genomic rescue strategies may prove useful for re-evaluating other long-standing *Drosophila* developmental models.

## Introduction

*eyes absent (eya)* encodes a highly conserved transcriptional coactivator and protein phosphatase whose homologs play vital roles in human development [1], [2], [3], [4]. Mutations in human *EYA1* lead to the autosomal dominant disorder known as branchio-oto-renal (BOR) syndrome, characterized by craniofacial anomalies, hearing loss, and kidney defects [5]. In addition, *EYA* overexpression occurs in a number of human solid tissue tumors, and correlates with poor prognosis in breast cancer [6], [7], [8], [9].

*Drosophila melanogaster* has a single *eya* gene, which is essential for survival [1], [10]. Eya regulates development of the gonads, muscle, and the eye, where it has been most extensively studied [1], [11], [12], [13]. The compound eye of adult *Drosophila* arises from a larval epithelial structure known as the eye imaginal disc. The eye disc initially consists of undifferentiated, proliferating cells. Later, at the onset of the third instar larval stage, an indentation called the morphogenetic furrow forms at the posterior margin of the eye disc and sweeps toward the anterior margin, triggering the onset of differentiation [14]. Eya expression begins in the eye disc during the second instar stage, prior to furrow initiation. Once the furrow starts, Eya continues to be expressed in a domain anterior to the furrow as well as in differentiating cells in the posterior part of the disc [1]. Eye discs that lack *eya* begin to develop normally, but the furrow fails to initiate, differentiation does not occur, and the eye disc undergoes widespread apoptosis, resulting in complete loss of the adult eye [1]. *eya* also appears to be required for differentiation or survival of photoreceptor cells behind the furrow [15]. Hence, understanding the regulation of Eya function may provide essential insight into the mechanisms of eye development.

Eya is a member of the retinal determination (RD) network, a small group of highly conserved transcriptional regulators that are both necessary for eye development and sufficient to trigger ectopic eye formation when overexpressed in other imaginal discs. Other key members of the RD network include Eyeless, Sine oculis, and Dachshund [16], [17], [18], [19]. Eye development involves complex regulatory interactions among the RD members as well as signaling pathways [20], [21], [22], [23], [24]. While the regulation of RD genes has been studied extensively at the level of transcription [25], [26], [27], [28], [29], relatively little is known about the role of post-translational modification in regulating RD factors.

Previous studies have suggested that one mechanism of post-translational regulation of Eya activity in the eye is through phosphorylation by mitogen activated protein kinases (MAPK). Two Eya residues, S402 and S407, strongly match the MAPK target motif [30]. These residues have been shown to undergo phosphorylation in vitro by the MAPK family kinases Erk and Nemo (Nmo) [31], [32]. Using ectopic eye induction as an assay of Eya activity, these studies have suggested that phosphorylation by Erk and Nmo at these residues activates Eya [31], [32]. A transgene encoding a protein that cannot be phosphorylated at these residues (*UAS-eya^SA^*) shows a lower frequency of ectopic eye induction compared with a wild-type *UAS-eya* transgene. In contrast, *UAS-eya^SDE^*, which encodes a phosphomimetic protein, induces ectopic eyes more frequently than wild-type *UAS-eya* [31]. Genetic interaction studies, likewise using ectopic eye induction, have supported this model. Specifically, co-overexpression of *eya* with a hyperactive allele of *rolled* (*rl*, which encodes Erk) or with *nmo* leads synergistically to more and larger ectopic eyes. Conversely, loss of one copy of *rl* or *nmo* leads to weaker ectopic eye induction by *eya* [31], [33]. *UAS-eya^SA^* also loses the ability to synergize with *UAS-nmo* in ectopic eye induction [32]. Together, these results have led to the currently accepted model that MAPK-mediated phosphorylation of Eya on S402 and S407 positively regulates Eya in development.

The above studies all utilized ectopic overexpression assays, in which the cDNA for the gene of interest is expressed in a defined spatiotemporal domain using the *Gal4/UAS* system [34]. This approach has yielded many insights into the regulatory relationships among genes, as well as helping discern the in vivo function of protein domains and motifs [16], [19], [26], [35], [36], [37], [38]. However, *Gal4/UAS* assays face certain limitations: the levels of transgene expression differ from those of the endogenous gene, and random integration of the transgene into the genome leads to position effects, which make direct comparison of distinct transgene lines problematic. Moreover, not all findings of an ectopic expression experiment may be applicable to normal development, where the gene of interest acts in a different cellular context and in the presence of different binding partners and signaling pathways that may affect its function. More recently, the development of genomic rescue transgenes has made it possible to analyze the function of protein domains and motifs in the context of normal development [39]. However, to date, genomic rescue has not yet been employed to verify the native function of genes previously defined in ectopic studies. For these reasons, we sought to analyze the function of the MAPK target residues of Eya in the context of normal rather than ectopic eye development, using a genomic rescue strategy.

In the current study, we generate a series of *eya* genomic rescue constructs that fully rescue eye development, as well as all other known *eya* mutant phenotypes. Surprisingly, we find that in contrast to the effect of Eya phosphorylation on ectopic eye induction [31], [32], neither loss of MAPK target sites S402 and S407 nor phosphomimetic mutations at these sites affects normal eye development or survival. Our study is the first example of a genomic rescue system yielding results different from those of a cDNA-based ectopic overexpression assay, and underscores the importance of studying a gene in its native context.

## Results

### The transgene *eya^+^GR* rescues *eya* mutant phenotypes

**Figure 1:**
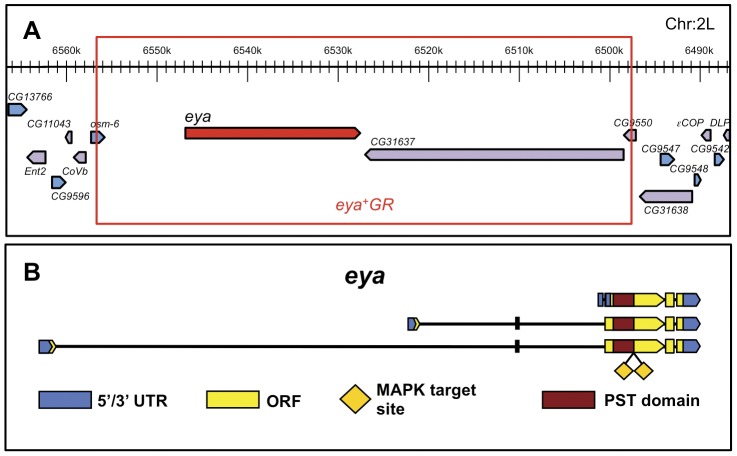
Construction of *eya^+^GR* and point mutations. **A**. Schematic of the *eya^+^GR* transgene (adapted from http://flybase.org). The red box indicates the extent of the 58.8 kb genomic rescue fragment, which includes the entire *eya* gene, as well as the upstream sequence up to the nearest gene (*osm-6*) and the entire gene that lies immediately downstream of *eya*, *CG31637*. *CG31637* encodes a predicted sulfotransferase and has no known mutant phenotypes. The transgene was made in *attB-P[acman]-Ap^R^* (see Materials and Methods). **B**. Schematic of the *eya* gene. Yellow diamonds indicate the MAPK target sites S402 and S407, both of which are in the PST (proline-serine-threonine rich) transcriptional coactivator domain of Eya, indicated by the maroon rectangle.

Prior evidence that MAPK-mediated phosphorylation activates Eya came from ectopic eye induction studies, which relied on *Gal4/UAS*-mediated overexpression of cDNA-based *eya* transgenes [31], [32]. To investigate how phosphorylation regulates Eya function during normal eye development, we generated genomic rescue transgenes, which offer two key advantages over the *Gal4/UAS* system. First, the genomic transgene contains regulatory sequences that drive expression of the gene of interest in a wild-type pattern and at levels matching those of the native gene. Second, the transgene is inserted in a specific site in the genome, allowing comparison of independent transgenic lines without confounding position effects [39]. We made *eya^+^GR* (Genomic Rescue), a 58.8 kb fragment encompassing the *eya* gene and flanking regions, in the *P[acman]* vector using recombineering [39] (Fig. 1). We used a large genomic rescue fragment to increase the chances of including regulatory regions necessary for all *eya* expression, as *eya* enhancers are currently not fully characterized. The transgene was introduced into *P2*, a specific and reproducible “*attP*” insertion site on the third chromosome [39].

**Figure 2:**
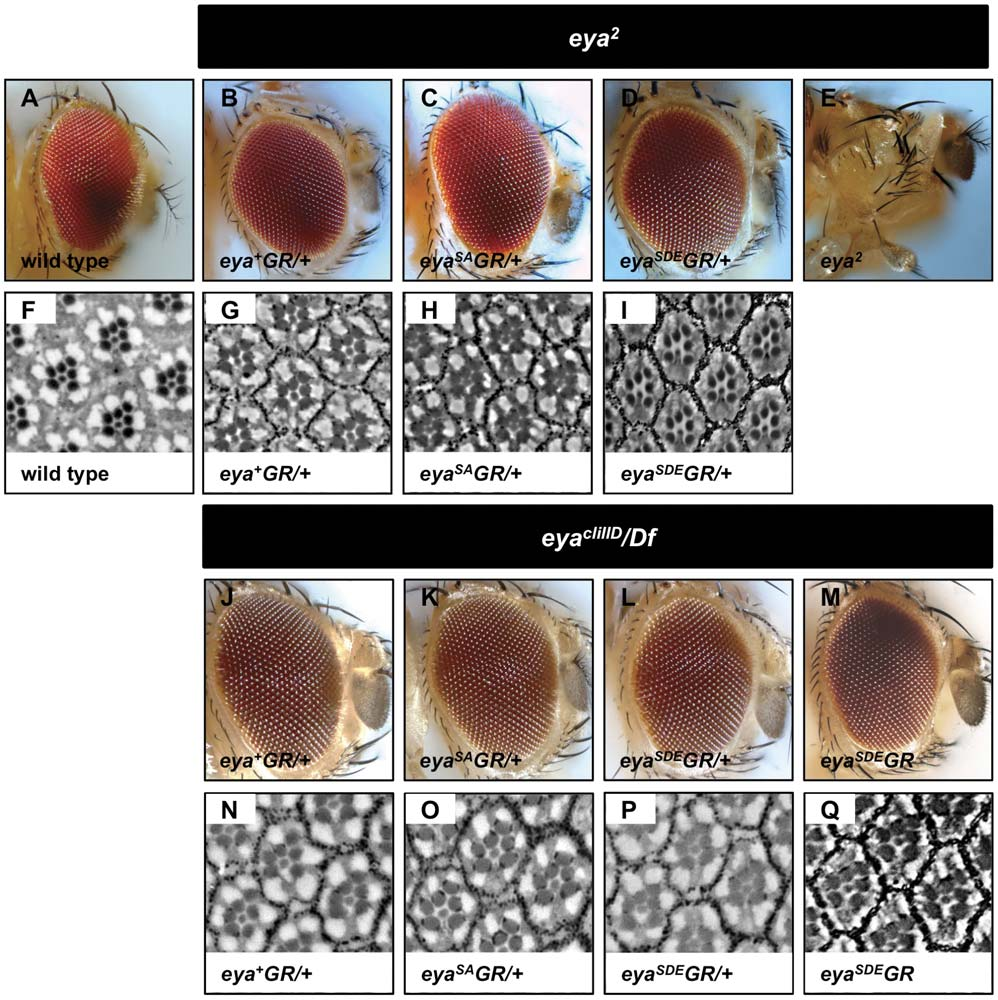
*eya^+^GR*, *eya^SA^GR*, and *eya^SDE^GR* rescue eye development and survival in *eya* mutants. External (A–E, J–M) and internal (F–I, N–Q) adult eye morphology is fully rescued with genomic transgenes in *eya* mutants. Homozygous *eya^2^* adults carrying a single copy of *eya^+^GR* (B, G), *eya^SA^GR* (C, H), or *eya^SDE^GR* (D, I) have eye morphology indistinguishable from wild type Canton S (A, F), whereas *eya^2^* homozygotes without a rescue construct show complete loss of the compound eye (E). *eya^cliIID^/Df* flies die due to lack of all endogenous *eya* function, and one copy of *eya^+^GR* (I, M), *eya^SA^GR* (J, N), or *eya^SDE^GR* (K, O) restores viability and normal eye morphology. Two copies of *eya^SDE^GR* (M, Q) rescue viability and eye morphology in *eya* null adults equally well as one copy of *eya^SDE^GR*, indicating no gain-of-function phenotype due to the phosphomimetic Eya^SDE^ protein.

We tested the ability of *eya^+^GR* to rescue eye development in *eya^2^* mutants. *eya^2^* has a deletion of an eye-specific enhancer, leading to loss of *eya* expression only in the eye disc. Consequently, *eya^2^* homozygous adults are viable and fertile, but completely lack eyes [1], [40]. One copy of *eya^+^GR* rescues eye formation in *eya^2^* homozygotes. The eyes of *eya^2^; eya^+^GR/+* flies are indistinguishable from wild type by external morphology and size, and sections show wild-type arrangement and number of rhabdomeres per ommatidium (Fig. 2).

**Figure 3:**
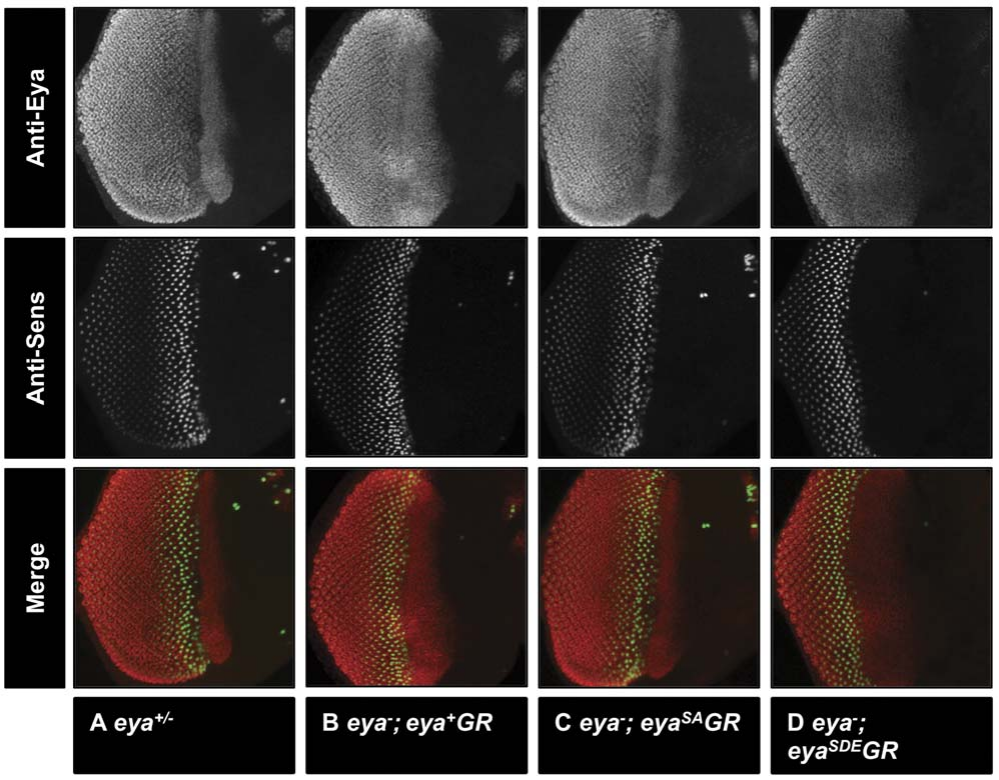
*eya^+^GR*, *eya^SA^GR*, and *eya^SDE^GR* rescue larval eye disc development. Late third instar eye imaginal discs from *Df/+* (A), *eya^cliIID^/Df; eya^+^GR/+* (B), *eya^cliIID^/Df; eya^SA^GR/+* (C), and *eya^cliIID^/Df; eya^SDE^GR/+* (D) larvae have been stained with anti-Eya (A–D) and anti-Sens to mark differentiating R8 photoreceptors (E–H); merged images shown in I–L. Eya expression pattern and levels are similar between heterozygous larvae and null *eya* larvae rescued with one copy of each *eya* transgene. Differentiation occurs normally in rescued eye discs.

**Figure 4:**
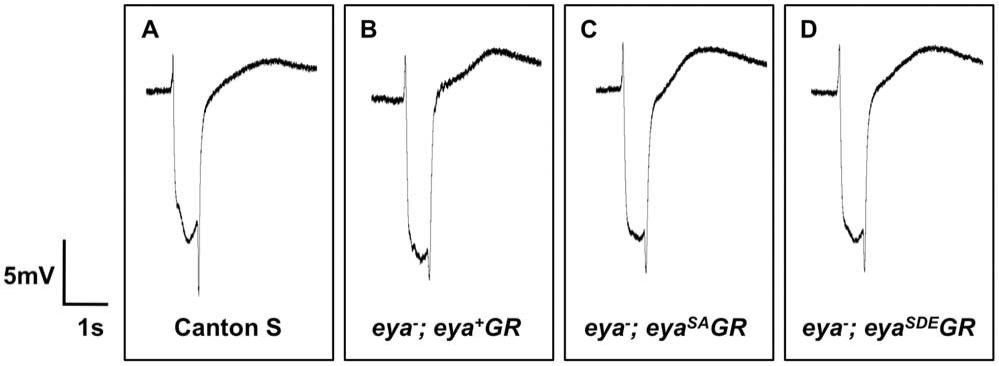
Animals rescued with eya*GR have normal eye function. Representative ERG traces of Canton S (wild-type) (A), *eya^cliIID^/Df; eya^+^GR/+* (B), *eya^cliIID^/Df; eya^SA^GR/+* (C), and *eya^cliIID^/Df; eya^SDE^GR/+* (D) adults. *eya* null adults rescued with a single copy of *eya^SA^GR* or *eya^SDE^GR* show ERG responses indistinguishable from flies rescued with *eya^+^GR* or wild-type flies.

**Figure 5:**
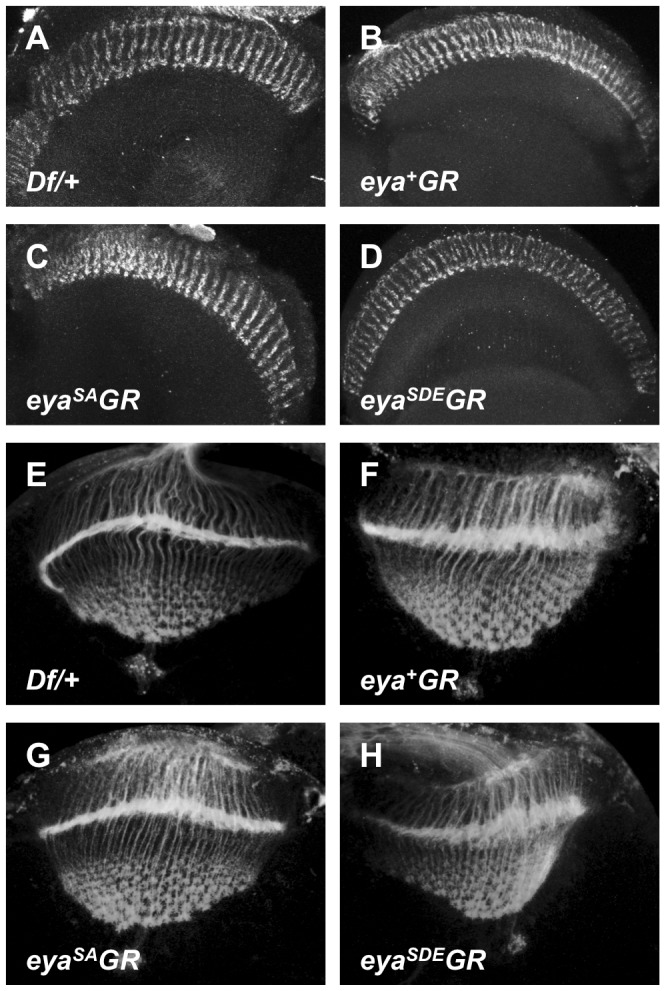
Photoreceptor axon projections are normal in flies rescued with eya*GR. Photoreceptor axon projections to the optic lobe of *Df/+* (A, E), *eya^cliIID^/Df; eya^+^GR/+* (B, F), *eya^cliIID^/Df; eya^SA^GR/+* (C, G), and *eya^cliIID^/Df; eya^SDE^GR/+* (D, H) are indistinguishable from each other in both adults (A–D) and wandering third instar larvae (E–H). Axon projections are visualized with anti-Chaoptin (24B10).

We also tested whether *eya^+^GR* can rescue *eya^cliIID^*, a null allele that results in embryonic lethality [10]. *eya^cliIID^* fails to complement *Df(2L)BSC354* (hereafter referred to as *Df*), a molecularly defined deficiency [41] that uncovers *eya*. One copy of *eya^+^GR* fully rescues the lethality of *eya^cliIID^/Df* flies, and the adult eyes of rescued flies are indistinguishable from wild-type, both by external morphology and in sections (Fig. 2). We refer to *eya^cliIID^/Df; eya^+^GR/+* flies hereafter as *eya^−^; eya^+^GR* flies. The late third instar larval eye discs of *eya^−^; eya^+^GR* are indistinguishable from wild type eye discs in size and morphology, and immunohistochemistry reveals similar Eya levels and expression patterns (Fig. 3). Likewise, differentiation proceeds normally, as shown by the R8 photoreceptor marker Senseless (Sens) (Fig. 3). *eya^−^; eya^+^GR* flies are present at Mendelian ratios (Table 1), indicating that the rescued flies do not have a survival disadvantage compared with their *eya^cliIID^/CyO* or *Df/CyO* siblings. Hence, a single copy of the *eya^+^GR* transgene is functionally equivalent to the single copy of endogenous wild-type *eya* on the *CyO* chromosome of *eya^−^/CyO* flies. We also tested the function of rescued eyes using electroretinograms (ERG) and found no difference between wild-type (Canton S) and *eya^−^; eya^+^GR* flies (Fig. 4). In addition, Eya regulates photoreceptor axon targeting to the brain [42]. We analyzed photoreceptor axon projections in *eya^−^; eya^+^GR* adults and third instar larvae, and found no difference in axon projections compared with *Df/CyO* (Fig. 5). Altogether, these observations indicate that one copy of our wild-type rescue transgene behaves similarly to an endogenous copy of *eya*.

**Table 1:** eya*GR fully rescues viability in *eya^−^* flies.

Genotype	*Cy* obs.	*Cy* exp.	Non-*Cy* obs.	Non-*Cy* exp.	Total	?^2^
*eya^+^GR*	278	273	131	136	409	0.28
*eya^SA^GR*	216	223	119	112	335	0.66
*eya^SDE^GR*	261	259	127	129	388	0.046

Progeny from w; eya^cliIID^/CyO; eya*GR×*w; Df(2L)BSC354/CyO* cross are present at Mendelian ratios, indicating that *eya^−^* flies rescued with a single copy of *eya^+^GR*, *eya^SA^GR*, or *eya^SDE^GR* do not have a survival disadvantage compared with *eya^−^/CyO* siblings. χ^2^ critical (1 d.f. P 0.05) = 3.84.

**Figure 6:**
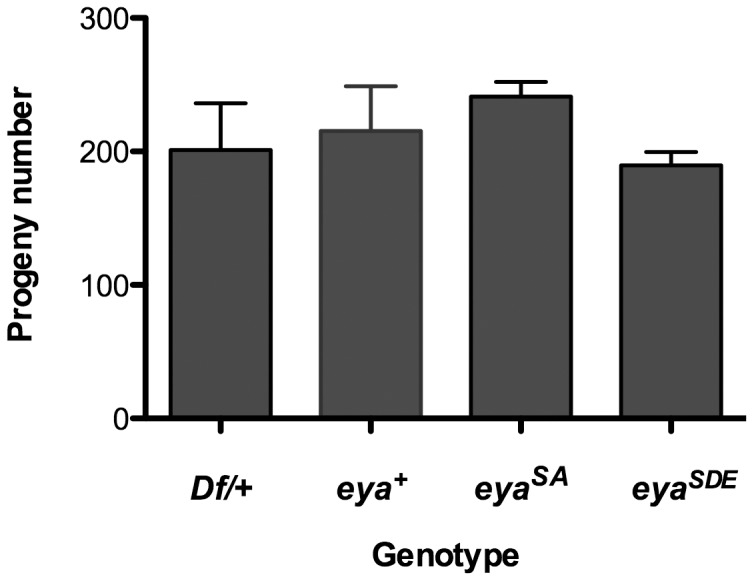
eya*GR rescues fertility in *eya* null males. The numbers of progeny are not statistically different (P = 0.156) among *w* virgin females crossed to *Df/CyO* control, *eya^−^; eya^+^GR*, *eya^−^; eya^SA^GR*, and *eya^−^; eya^SDE^GR* males. Error bars indicate standard deviation. n = 3 crosses for each genotype.

Since Eya is required for somatic gonad development, and a partial loss-of-function *eya* allele causes male and female sterility [11], [12], [43], we tested whether *eya^+^GR* rescues fertility in *eya^cliIID^* homozygotes. *eya^cliIID^; eya^+^GR* females are fertile (data not shown), and *eya^+^GR* rescued males produce indistinguishable numbers of progeny from *eya* heterozygous control males when crossed to *w* virgin females (Fig. 6). In addition to being required for survival and for eye and gonad development, *eya* regulates muscle development [13]. The *eya^cliIID^; eya^+^GR* adults appear to move normally and are able to fly (data not shown), indicating that gross muscular defects in these flies are unlikely. In summary, we conclude that the *eya^+^GR* construct fully rescues all known aspects of *eya* function in *Drosophila*, and provides a critical tool for examining the role of specific Eya residues in normal eye development and survival.

### The phosphorylation state of Eya residues S402 and S407 does not affect eye morphology or survival

Having found that *eya^+^GR* rescues *eya* loss-of-function phenotypes, we next generated two point-mutant genomic rescue constructs to investigate the role of Eya phosphorylation during normal eye development. Eya residues S402 and S407 are two serines that undergo Erk- and Nmo-mediated phosphorylation [31], [32]. In the first point-mutant genomic construct, *eya^SA^GR*, we mutated S402 and S407 to alanines, which cannot be phosphorylated. Conversely, in the second point-mutant genomic construct, *eya^SDE^GR*, we mutated S402 and S407 to aspartate and glutamate, respectively, mimicking constitutive phosphorylation. We integrated both constructs into the same *attP* site as *eya^+^GR* in order to avoid differences among the rescue phenotypes due to position effects (see Materials and Methods).

Based on previous data [31], we expected that *eya^SA^GR* would result in partial or no rescue of eye formation in *eya* mutants, and that *eya^SDE^GR* would lead either to full rescue or to a phenotype partially resembling Eya overexpression in the eye. Unexpectedly, both *eya^SA^GR* and *eya^SDE^GR* (hereafter referred to collectively as eya*GR) rescue eye formation in *eya^2^* homozygotes to the same extent as *eya^+^GR*. The external morphology of eya^2^; eya*GR/+ eyes is indistinguishable from *eya^2^; eya^+^GR/+* eyes (Fig. 2). Sections reveal the normal number and arrangement of photoreceptors in eya^2^; eya*GR/+ eyes (Fig. 2).

We also tested the ability of eya*GR to restore survival and eye formation to *eya* null mutants. eya^−^; eya*GR adults are viable and present at Mendelian ratios (Table 1). Moreover, the eyes of eya^−^; eya*GR adults appear wild-type in external morphology and in sections (Fig. 2). Because a single copy of eya*GR appears to be functionally equivalent to one copy of endogenous *eya* in restoring viability, we performed the remaining experiments on *eya* null flies with one copy of eya*GR. Since *eya^SDE^GR* encodes a phosphomimetic function that may act as a hypermorph, we considered the possibility that *eya* null flies rescued with two copies of *eya^SDE^GR* may display gain-of-function phenotypes not seen in flies with one copy of *eya^SDE^GR*. However, *eya^cliIID^* homozygous adults with two copies of *eya^SDE^GR* have the same external and internal eye morphology as null flies with a single copy of eya*GR (Fig. 2).

Based on the adult eye phenotypes of eya^−^; eya*GR flies, we expected to observe normal Eya expression in the developing eye discs of eya^−^; eya*GR larvae. Immunohistochemistry reveals similar Eya levels and expression patterns among *Df/+*, *eya^−^; eya^+^GR*, and eya^−^; eya*GR late third instar larval eye discs (Fig. 3). This is consistent with previous findings that the SA and SDE mutations do not affect Eya stability [31]. Loss of *eya* from the eye disc leads to a failure of differentiation and to widespread apoptosis, which causes a severe reduction in eye disc size in the third instar [1]. By contrast, the eye discs of eya*GR-rescued flies are indistinguishable from *eya^−^; eya^+^GR* and wild type eye discs in size and morphology, and differentiation proceeds normally, as shown by the R8 photoreceptor marker Senseless (Sens) (Fig. 3).

In addition to restoring survival and eye development, eya*GR transgenes also rescue fertility and muscle development. We observed that eya^cliIID^; eya*GR females are fertile (data not shown), and we quantified the number of progeny produced by eya*GR rescued males crossed to *w* virgin females (Fig. 6). eya*GR rescued males produce statistically the same numbers of progeny as males rescued with *eya^+^GR* and *eya* heterozygous males (Fig. 6). Similarly to *eya^−^; eya^+^GR* adults, the eya^−^; eya*GR adults appear to move normally, making it unlikely that S402 and S407 phosphorylation regulates Eya in muscle development (data not shown). We conclude that, in contrast to previous findings using ectopic eye induction as an assay [31], MAPK-mediated phosphorylation of S402 and S407 does not regulate Eya during normal *Drosophila* development.

### Phosphorylation of Eya residues S402 and S407 is not required for eye function

Electroretinogram (ERG) assays indicate that normal response to light is rescued in eya^−^; eya*GR eyes (Fig. 4). In addition, we analyzed photoreceptor axon projections to the adult brain in eya^−^; eya*GR flies. Since tyrosine phosphorylation of Eya by the Abelson kinase regulates photoreceptor axon projections [42], we asked whether Eya phosphorylation by MAPK might also play a role in this process. However, we found no difference in axon projections between eya^−^; eya*GR and *eya^−^; eya^+^GR* adults (Fig. 5). Likewise, *eya* null third instar larvae rescued with a copy of eya*GR show an even pattern of axon projections at the lamina of the optic lobe that is the same as in *eya* heterozygotes, rather than the irregular gaps and thickenings in the lamina plexus previously reported in *eya* loss-of-function mutant larvae [42]. We conclude that phosphorylation of Eya at S402 and S407 is not required for eye development or function.

## Discussion

*Drosophila eyes absent (eya)* is essential for survival [1], [10] and is required for eye development, as well as regulating development of the gonads and muscles [1], [11], [12], [13]. Previous studies using ectopic overexpression of *eya* cDNA transgenes during the past decade have suggested that phosphorylation by MAPK may activate Eya during *Drosophila* eye development [31], [32]. Similar to the *Drosophila* studies, a recent report in mice showed that loss of conserved MAPK target sites in murine Eya1 reduced its ability to induce ectopic hair cell formation in the cochlea [44]. These cumulative ectopic expression studies in multiple species have led to the current model that Eya function is activated by phosphorylation at specific, conserved MAPK target sites. However, in contrast to these ectopic studies, we show that both the genomic rescue transgene *eya^SA^GR*, which leads to loss of phosphorylation on two MAPK target residues, and *eya^SDE^GR*, which encodes a protein with phosphomimetic amino acids at the same two residues, rescue normal eye development and survival in *eya* mutants as effectively as the wild-type *eya^+^GR* transgene. While we cannot rule out subtle phenotypes, overall our data indicate that phosphorylation of Eya on S402 and S407 does not regulate Eya function during normal development.

### Differences between ectopic eye induction vs. normal eye development

The ability of *eya^SA^GR* and *eya^SDE^GR* to rescue eye development may be explained by the difference in context between the eye disc and other discs being reprogrammed to form ectopic eyes. Overexpression of a retinal determination network (RD) transgene such as *eya* can trigger ectopic eye formation only in retinal “hot spots”, small subsets of cells in the antenna, leg, wing, and haltere imaginal discs [45]. Some of these “hot spots” overlap with sites capable of transdetermination (a process in which one type of imaginal disc assumes the fate of another, in response to injury or genetic manipulation) [46], [47]. This suggests that ectopic eye induction is confined to populations of cells that have a high level of developmental plasticity, perhaps due to their chromatin state or the activity of signaling pathways [45]. Transforming these cell populations into ectopic retinal tissue may require different factors from those needed for normal eye development.

For example, *sine oculis (so)* encodes a transcription factor in the RD network that is both necessary and sufficient for eye development [18], [48]. A *UAS* transgene encoding a constitutively repressive form of So can still induce ectopic eyes in the antenna, but it cannot rescue the loss of the normal eye in *so^1^* mutants. Conversely, a *UAS* transgene encoding So fused to a strong transcriptional activator domain fails to trigger ectopic eye formation, but it restores normal eye development in *so^1^* mutants. These results indicate different requirements for transcriptional repression vs. activation by So in ectopic and normal eye formation [49]. In another example of a difference between ectopic and normal eye development, the kinase Nmo synergizes with Eya to induce ectopic expression of *dachshund* (*dac*) and *lozenge* (*lz*), yet neither *dac* nor *lz* expression is affected in *nmo* loss-of-function clones in the eye [32], [33].

Taken together, our results and previous studies [31], [32] indicate that the function of Eya phosphorylation differs between ectopic and normal eye development. One explanation may be that a higher level of Eya activity is needed to reprogram antenna into eye, compared with the level required for normal eye formation. Alternatively, phosphorylation may improve Eya's ability to regulate a target (or targets) in the antenna that is not normally expressed in the eye disc or relevant to normal eye development.

### Transgene position effects in previous ectopic eye studies

In addition to the differences between normal and ectopic eye development, the transgenic systems used in previous studies vs. the current study may have contributed to the difference in results as well. The previous study [31] tested the effect of phosphorylation on Eya function using *UAS-eya* transgenes that integrated randomly in the genome. While the average efficiency of eye induction was higher for *UAS-eya^+^* (49%) than for *UAS-eya^SA^* (19%), and lower for *UAS-eya^+^* than for *UAS-eya^SDE^* (81%), these averages do not reveal the large differences among lines expressing the same transgene. Among eight independent *UAS-eya^+^* transgenic lines, the frequency of ectopic eye induction ranged from 3% to 78% [31], suggesting considerable position effects. In contrast, we used site-specific integration of *eya* genomic rescue transgenes, which is expected to minimize the differences among transgenic lines due to position effects.

### Alternative mechanisms for Eya regulation

While our results indicate that S402 and S407 of Eya are not required for survival or eye formation, we do not rule out the possibility that Erk and/or Nmo regulates Eya in the eye by phosphorylation on other serine or threonine residues. S402 and S407 are the only Eya residues that match the “strong” consensus MAPK target site, but 14 additional previously unstudied Eya residues match a less stringent MAPK consensus [30]. Ten of these “weak” MAPK consensus sites reside along with S402 and S407 in the Eya proline-serine-threonine rich (PST) domain, which is robustly phosphorylated by Erk and Nmo in vitro. While phosphorylation of S402 and S407 appears to account for approximately 80% of Nmo-mediated and more than 90% of Erk-mediated phosphorylation of the Eya PST domain in vitro, when S402 and S407 are both mutated to alanines, the point-mutant PST domain can still be weakly phosphorylated in vitro by MAPK [31], [32]. Whereas Nmo appears to phosphorylate only the N-terminal part of Eya, which includes the PST domain, rather than the C-terminal domain [32], the ability of Erk to phosphorylate Eya C-terminal domain has not been assayed. It remains to be tested which Eya residues besides S402 and S407 can be phosphorylated by Erk and/or Nmo, and whether such phosphorylation regulates Eya in vivo. The observed interaction between Eya and MAPK might also be due to MAPK-mediated phosphorylation of the Eya binding partner and transcription factor So, which undergoes serine/threonine phosphorylation in cell culture [50]. Future studies, perhaps using mass spectrometry, will be needed to elucidate the extent of Eya and/or So phosphorylation by MAPK.

The investigation of MAPK-mediated phosphorylation of Eya was prompted by the finding that *eya* interacts genetically with the Egfr pathway in the eye [51]. Activation of the Egfr pathway in the eye leads to activation of the MAPK Erk, which can then phosphorylate Eya (reviewed by [52]). However, if MAPK does not regulate Eya by phosphorylation, the genetic interaction between *eya* and the Egfr pathway may be due to transcriptional activation of *eya* by Egfr signaling. A recent study has shown that Egfr signaling regulates *eya* expression in both the eye disc and the embryo, and that loss of *pointed*, which encodes a transcription factor that acts downstream of Egfr, causes strong reduction in Eya expression in eye disc clones [53].

In summary, Eya and its homologs play an essential role in regulating multiple aspects of development from *Drosophila* to humans. A long-standing model of Eya regulation, based on ectopic eye induction assays, has posited that MAPK-mediated phosphorylation at residues S402 and S407 potentiates Eya activity [31], [32]. Using genomic rescue transgenes, we demonstrate that unlike ectopic eye development, normal eye formation and survival are unaffected by either loss of phosphorylation or phosphomimetic mutations at the two previously studied MAPK target residues of Eya. This is the first study to use a genomic rescue approach in *Drosophila* to reassess the biological relevance of ectopic overexpression studies of the same gene. These findings indicate similar genomic rescue strategies may prove useful for re-evaluating other *Drosophila* developmental models.

## Materials and Methods

### Construction of *eya^+^GR*

A 58.8 kb fragment encompassing the *eya* gene and flanking regions was cloned into the *attB-P[acman]-Ap^R^* vector using recombineering as described previously [39]. The resulting construct was end-sequenced and integrated into the *P{CaryP}attP2* site (abbreviated *P2*), which is located at 3L:11,063,638, using φC31 integrase [54]. Site-specific integration was confirmed by PCR with *attB/attP* primers [39].

### Recombineering-induced point mutagenesis of *eya^+^GR*

The codons encoding S402 and S407 (TCC and TCG, respectively) in *eya^+^GR* were mutated to GCC and GCG to make *eya^SA^GR* and to GAC and GAG to make *eya^SDE^GR*. We used two-step recombineering with the *catSacB* cassette, which provides positive selection (*cat*, chloramphenicol resistance) and negative selection (*SacB*, sucrose sensitivity). The protocol was performed as described previously [55]. Since S402 and S407 are only five amino acid residues apart, both codons were targeted in one recombineering event. Putative recombinants were tested by sequencing, and true positives were tested for rearrangements by restriction digest fingerprinting. Both point mutant transgenes were injected into *P2*, the same site used for *eya^+^GR*, and site-specific integration was verified by PCR with *attB/attP* primers [39]. The presence of point mutations was verified by restriction digest of genomic PCR products. Both S407A and S407E mutations create a *Pvu*I site restriction enzyme site (CGATCG) that is not present in wild-type *eya* (CGATCT). We performed PCR on genomic DNA from wild-type, *eya^+^GR*, *eya^SA^GR*, and *eya^SDE^GR* adults using *MAPK1-for* and *MAPK2-rev* primers that flank the S402 and S407 codons and give a 1,058 bp product. The PCR product was digested with *Pvu*I and run on a gel. The enzyme cut the *eya^SA^GR* and *eya^SDE^GR* PCR product only, resulting in 500 and 558 bp bands. Primer sequences are available on request.

### Histology and imaging of adult eyes

Adult eye sections were performed as described previously [56]. Images of eye sections and whole adult eyes were taken with a Zeiss Axioplan 2 microscope and AxioVision software. Images of whole-mount adult eyes were processed with CZ Focus software. All images were further processed with Adobe Photoshop software.

### Immunohistochemistry of adult brains and 3^rd^ instar eye discs

Brains were dissected and stained as previously described [57] out of adults and late wandering third instar larvae. For larval eye disc dissections, w/Y; eya^cliIID^/BSC354; eya*GR/+ males were crossed with *w; BSC354/CyO, GFP* females and progeny larvae were scored for absence of GFP expression (w; eya^cliIID^/BSC354; eya*GR/+). Eye imaginal discs were dissected out of wandering third instar larvae in 1× PBS and fixed in 4% formaldehyde in PBS 20 minutes on ice. Discs were washed with PBS, PAXD (1× PBS with 1% BSA, 0.3% Triton X-100, and 0.3% sodium deoxycholate), and PAXDG (5% normal goat serum in PAXD) on ice, 10 minutes per wash. Discs were then incubated with primary antibody in PAXDG at 4°C overnight. Subsequent steps were at room temperature. The following day the discs were washed 3× with PAXDG, 10 minutes per wash, and incubated in secondary antibody in PAXDG 2 hours. The discs were washed with PAXDG, PAXD, and PBS, 10 minutes per wash, and post-fixed in 4% formaldehyde in PBS 15 minutes. The discs were then washed twice with PBS (first wash quick, second wash 10 minutes) and incubated in Vectashield (Vector Laboratories, Inc.). Primary antibodies used were 1∶100 mouse anti-Chaoptin (24B10, Developmental Studies Hybridoma Bank), 1∶200 mouse anti-Eya (10H6, Developmental Studies Hybridoma Bank) and 1∶100 guinea pig anti-Sens (gift from H. Bellen). Secondary antibodies used were Cy3 goat anti-mouse (1∶200 for brains, 1∶500 for discs; Jackson ImmunoResearch) and 1∶500 Alexa Fluor 488 goat anti-guinea pig (Molecular Probes). Images were taken with a Zeiss LSM 510 confocal microscope and processed with Image J and Adobe Photoshop software.

### Electroretinogram recordings

Electroretinograms were performed as described previously [58]. Six three-day-old adults were assayed for each genotype.

### Survival assay

w/Y; eya^cliIID^/CyO; eya*GR males were crossed to *w; Df(2L)BSC354/CyO, Kr-GFP* virgin females. Expected (Mendelian) progeny ratios were 2/3 *Cy* (*eya^cliIID^/CyO* and *BSC354/CyO*) and 1/3 non-*Cy* (*eya^cliIID^/BSC354*). Based on observed numbers of adult progeny, for *eya^+^GR*, χ^2^ calculated = 0.28. For *eya^SA^GR*, χ^2^ calculated = 0.66. For *eya^SDE^GR*, χ^2^ calculated = 0.046. χ^2^ critical (1 d.f. P 0.05) = 3.84. For all genotypes, χ^2^ calculated<χ^2^ critical. Hence, for all genotypes, observed progeny ratios are not significantly different from expected ratios.

### Male fertility assay

Five males of each genotype (*w/Y; Df(2L)BSC354/CyO* control, *w/Y; eya^cliIID^/Df(2L)BSC354; eya^+^GR/+*, *w/Y; eya^cliIID^/Df(2L)BSC354; eya^SA^GR/+*, and *w/Y; eya^cliIID^/Df(2L)BSC354; eya^SDE^GR/+*) were crossed to ten *w^1118^* virgin females in triplicate. The flies were allowed to lay eggs for three days before being removed. We counted progeny that eclosed between days 9 and 16 after setting the cross, and the results were analyzed with one-way ANOVA.
